# The Japanese Orthopedic Association Back Pain Evaluation Questionnaire (JOABPEQ) for low back disorders: a validation study from Iran

**Published:** 2012-11

**Authors:** Parisa Azimi, Sohrab Shahzadi, Ali Montazeri

**Affiliations:** ^*a*^Department of Neurosurgery, Shahid Beheshti University of Medical Sciences, Tehran, Iran.; ^*b*^Department of Mental Health, Iranian Institute for Health Sciences Research, ACECR, Tehran, Iran.

## Abstract

**Background:**

Lumbar Disc Herniation (LDH) and Lumbar Spinal Stenosis (LSS) are the most common diagnoses of low back and leg pain symptoms. The Japanese Orthopedic Association Back Pain Evaluation Questionnaire (JOABPEQ) is a measure of health related quality of life in these patients. This study aimed to cross-culturally translate and validate the JOABPEQ in Iran.

**Methods:**

This was a prospective clinical validation trial. The translation and cross-cultural adaptation of the original questionnaire were performed in accordance with the published guidelines. A total of 103 patients with LDH or LSS were asked to respond to the questionnaire at two time points: pre- and post-operation (pre- and post-operative assessments) and were followed up for 6 months. To evaluate the reliability the internal consistency was assessed using the Cronbach's alpha coefficient and validity was assessed using the convergent validity. Responsiveness to the clinical change also was assessed comparing patients’ pre- and postoperative scores.

**Results:**

The Cronbach’s alpha coefficients for the JOABPEQ at preoperative and postoperative assessments ranged from 0.71 to 0.81 indicating a good internal consistency for the questionnaire. Furthermore, the correlation of each item with its hypothesized subscale of the JOABPEQ showed satisfactory results suggesting that the items had a substantial association with the subscale representing the concept. Further analysis also indicated that the questionnaire was responsive to change (P less than 0.0001).

**Conclusions:**

In conclusion, the Iranian version of JOABPEQ performed well and the findings suggest that it is a reliable and valid measure of back pain evaluation among LDH and LCS patients.

**Keywords:**

Japanese Orthopedic Association Back Pain Evaluation Questionnaire, Low back pain, Postoperative


					Volume 4, Suppl. 1 Nov 2012
Publisher: Kermanshah University of Medical Sciences
URL: http://www.jivresearch.org
Copyright:
Congress of Iranian Neurosurgeons, 14-16 November 2012, Kermanshah, Iran
Paper No. 81
The Japanese Orthopedic Association Back Pain Evaluation
Questionnaire (JOABPEQ) for low back disorders: a
validation study from Iran
Parisa Azimi a,*
, Sohrab Shahzadi a
, Ali Montazeri b
a
Department of Neurosurgery, Shahid Beheshti University of Medical Sciences, Tehran, Iran.
b
Department of Mental Health, Iranian Institute for Health Sciences Research, ACECR, Tehran, Iran.
Abstract:
Background: Lumbar Disc Herniation (LDH) and Lumbar Spinal Stenosis (LSS) are the most common diagnoses of low
back and leg pain symptoms. The Japanese Orthopedic Association Back Pain Evaluation Questionnaire (JOABPEQ)
is a measure of health related quality of life in these patients. This study aimed to cross-culturally translate and
validate the JOABPEQ in Iran.
Methods: This was a prospective clinical validation trial. The translation and cross-cultural adaptation of the original
questionnaire were performed in accordance with the published guidelines. A total of 103 patients with LDH or LSS
were asked to respond to the questionnaire at two time points: pre- and post-operation (pre- and post-operative
assessments) and were followed up for 6 months. To evaluate the reliability the internal consistency was assessed
using the Cronbach's alpha coefficient and validity was assessed using the convergent validity. Responsiveness to the
clinical change also was assessed comparing patients' pre- and postoperative scores.
Results: The Cronbach's alpha coefficients for the JOABPEQ at preoperative and postoperative assessments ranged
from 0.71 to 0.81 indicating a good internal consistency for the questionnaire. Furthermore, the correlation of each
item with its hypothesized subscale of the JOABPEQ showed satisfactory results suggesting that the items had a
substantial association with the subscale representing the concept. Further analysis also indicated that the
questionnaire was responsive to change (P less than 0.0001).
Conclusions: In conclusion, the Iranian version of JOABPEQ performed well and the findings suggest that it is a
reliable and valid measure of back pain evaluation among LDH and LCS patients.
Keywords:
Japanese Orthopedic Association Back Pain Evaluation Questionnaire, Low back pain,
Postoperative
* Corresponding Author at:
Parisa Azimi: Department of Neurosurgery, Imam-Hossain Hospital, Shahid-Beheshti University of Medical Sciences, Tehran, Iran.
Email: Parisa.azimi@gmail.com, (Azimi P.).

				

